# Can intracranial time-of-flight-MR angiography predict extracranial carotid artery stenosis?

**DOI:** 10.1007/s00415-021-10876-0

**Published:** 2021-11-09

**Authors:** Filiz Osmanodja, Jan F. Scheitz, Jochen B. Fiebach, Ramanan Ganeshan, Kersten Villringer

**Affiliations:** 1 Center for Stroke Research Berlin (CSB), Charité-Universitätsmedizin Berlin, Corporate Member of Freie Universität Berlin, Humboldt-Universität Zu Berlin, and Berlin Institute of Health, Berlin, Germany; 2 Department of Neurology, Charité-Universitätsmedizin Berlin, Corporate Member of Freie Universität Berlin, Humboldt-Universität Zu Berlin, and Berlin Institute of Health, Berlin, Germany; 3 Department of Neuroradiology, Friedrich-Alexander-University Erlangen-Nürnberg, Schwabachanlage 6, 91054 Erlangen, Germany

**Keywords:** Magnetic resonance angiography, Stroke, Intracranial embolism, Carotid stenosis

## Abstract

**Objectives:**

Extracranial stenosis of the internal carotid artery (ICA) is an important cause of ischemic stroke and transient ischemic attack (TIA). It can be diagnosed using contrast-enhanced CT or MR angiography (MRA) as well as Doppler ultrasound. In this study, we assessed the diagnostic value of intracranial time-of-flight (TOF) MRA to predict extracranial ICA stenosis (ICAS).

**Methods:**

We retrospectively analyzed consecutive patients with acute ischemic stroke or TIA and middle- (50–69%) or high-grade (70–99%) unilateral extracranial ICAS according to NASCET criteria assessed by ultrasound between January 2016 and August 2018. The control group consisted of patients without extracranial ICAS. Intraluminal signal intensities (SI) of the intracranial ICA on the side of the extracranial stenosis were compared to the contralesional side on TOF-MRA source images. SI ratios (SIR) of contralesional:lesional side were compared between groups.

**Results:**

In total, 151 patients were included in the main analysis. Contralesional:lesional SIR in the intracranial C4-segment was significantly higher in patients with ipsilateral extracranial ICA stenosis (*n* = 51, median 74 years, 57% male) compared to the control group (*n* = 100, median 68 years, 48% male). Mean SIR was 1.463 vs. 1.035 (*p* < 0.001) for right-sided stenosis and 1.362 vs. 1.000 (*p* < 0.001) for left-sided stenosis. Receiver-operating characteristic curve demonstrated a cut-off value of 1.086 for right-sided [sensitivity/specificity 75%/81%; area under the curve (AUC) 0.81] and 1.104 for left-sided stenosis (sensitivity/specificity 70%/84%; AUC 0.80) in C4 as a good predictor for high-grade extracranial ICAS.

**Conclusions:**

SIR on TOF-MRA can be a marker of extracranial ICAS.

**Supplementary Information:**

The online version contains supplementary material available at 10.1007/s00415-021-10876-0.

## Introduction

Extracranial internal carotid artery (ICA) stenoses constitute common and relevant causes of ischemic stroke. Patients with middle- or high-grade stenosis (> 50%) are more likely to experience worse functional outcome and early stroke recurrence than patients with no or low-grade (< 50%) stenosis [1, 2]. Especially in patients with high-grade (70–99%) extracranial ICA stenosis (ICAS), timely intervention can prevent recurrent ischemic stroke [3]. Therefore, timely identification of ipsilateral symptomatic ICAS is of clinical relevance.

In clinical practice, Doppler ultrasound (US) is a commonly accepted and easily accessible method in diagnosing extracranial ICAS. However, it has its drawbacks as well, that are investigator and experience dependency. In most comprehensive stroke centers, CT angiography (CTA) is still the primary examination in the emergency setting in patients with suspected acute stroke because of its broad and fast availability. MRI is also increasingly used in clinical practice because of the positive proof of the infarct core using diffusion-weighted imaging (DWI). Standard stroke MRI protocols do not routinely include extracranial contrast-agent-based MR angiography (MRA), mainly to reduce investigation time, but also due to the potential risk for allergic reactions. Another risk, nephrogenic systemic fibrosis seems to occur less frequently with novel contrast agents [4]. Time-of-flight (TOF) MRA has limitations in accuracy compared to contrast-enhanced MRA (CE-MRA) and it generally tends to overestimate the degree of the stenosis [5–7].

Based on the principle of flow-related signal changes in TOF-MRA, our assumption was that a proximal extracranial ICAS results in a lower ipsilateral intracranial signal intensity (SI) in TOF-MRA [8]. We hypothesized that SIs of the intracranial ICA differ in patients with extracranial ICAS compared to healthy controls.

## Materials and methods

### Study population

**Fig. 1:**
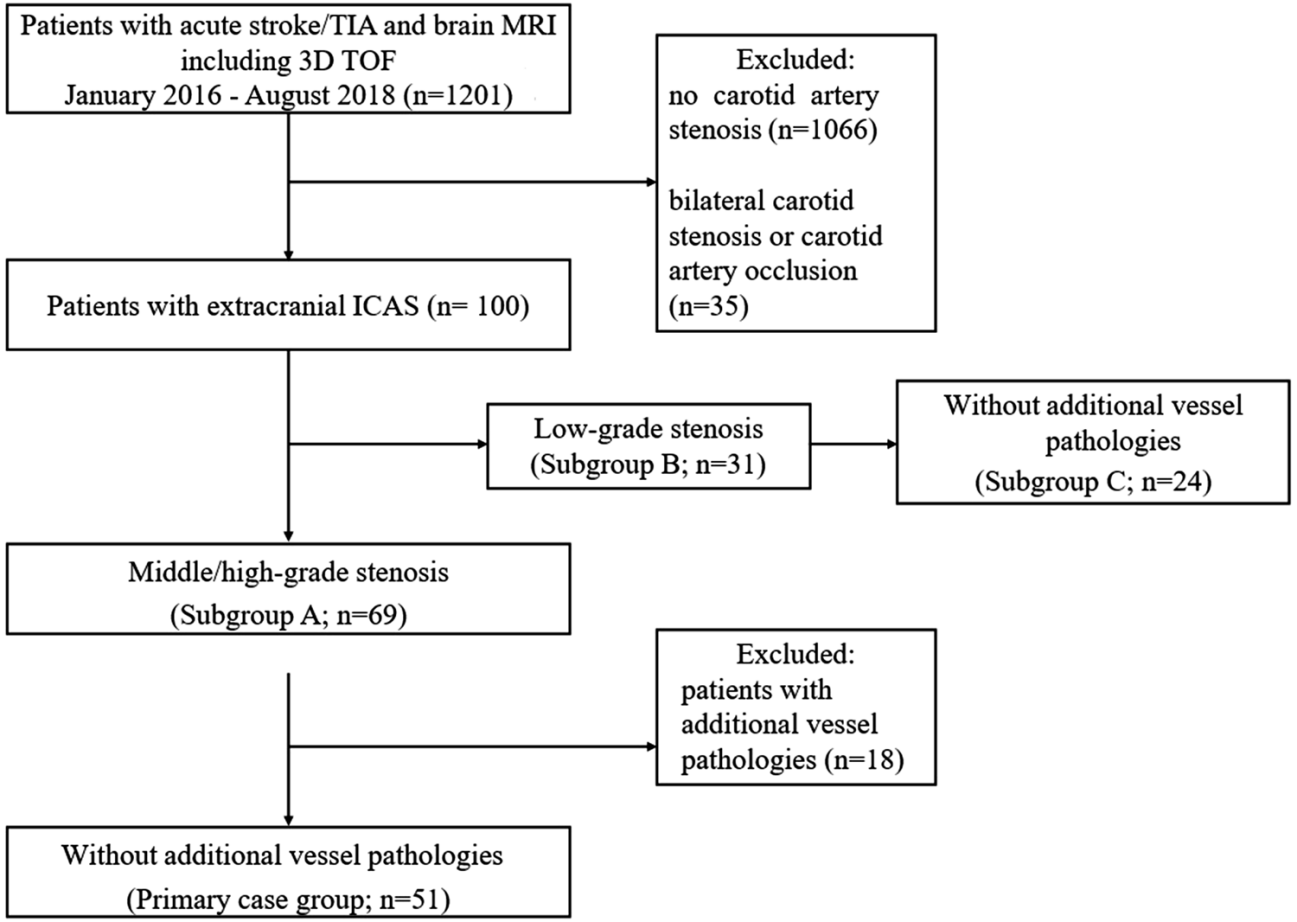
Flowchart of patient selection. TIA, transient ischemic attack. MRI, magnetic resonance imaging. TOF, time-of-flight. ICAS, internal carotid artery stenosis. Low-grade stenosis defined as < 50%, middle-grade stenosis as 50–69% and high-grade stenosis as ≥ 70% according to NASCET criteria [10]

We conducted a retrospective case–control study with consecutive patients with acute stroke or transient ischemic attack (TIA) between January 2016 and August 2018 at the Charité-Universitätsmedizin Berlin, who were examined on a 3 T MRI (Magnetom Trio; Siemens AG, Germany, 32-channel head coil) using a standard stroke protocol [9]. All patients were identified by searching our hospital digital patient records (SAP Clinical Workstation, SAP, Germany). Inclusion criterion was presence of a middle- (50–69%) or high-grade (> 70%) extracranial ICA stenosis (ICAS) according to the North American Symptomatic Carotid Endarterectomy Trial (NASCET) criteria [10] detected by ultrasound. Exclusion criteria were extracranial arterial occlusion or bilateral carotid stenosis regardless of the stenosis grade, as well as intracranial vessel pathologies (defined as: intracranial aneurysm, intracranial stenosis of the ICA in the C3–C7 segment, M1 stenosis) detected either by US, CE-MRA, or TOF-MRA. This resulted in *n* = 51 for the main analysis. The control group (*n* = 100) was formed by randomly selecting patients with suspected ischemic stroke or TIA without extracranial ICAS. Controls underwent the same MRI protocol during the same time period. Figure 1 shows a flowchart of patients’ selection. We conducted a secondary analysis including all patients with middle- or high-grade stenosis including those with vessel pathologies as defined above (subgroup A; *n* = 69). Subgroup B and C contained patients with low-grade stenosis (< 50%; according to NASCET criteria [10]) including (*n* = 31) and excluding those with vessel pathologies (*n* = 24), respectively.

Furthermore, patient’s characteristics, such as age, sex, history of cardiovascular risk factors (hypertension, dyslipidemia, diabetes mellitus, peripheral arterial occlusive disease, smoking history, and atrial fibrillation), and occurrence of a stenosis-related acute event (stroke or TIA), were documented. We also analyzed the etiology of stroke according to the classification of the Trial of ORG 10172 in Acute Stroke Treatment (TOAST) [11]; see supplementary data Table S3. In accordance with the Berlin State legislation, no separate ethics committee approval was required for this retrospective analysis.

### Imaging acquisition and analysis

All MRI examinations were performed on a 3 T MR scanner. The MRI standard stroke protocol contained DWI (slice thickness 2.5 mm, repetition time TR 8900 ms, echo time TE 93 ms, slice gap 0%, b values were 0 and 1000 mm × 2/s, 6 directions), T2*-weighted imaging (slice thickness 5 mm, TR 620 ms, TE 20 ms, slice gap 10%), 3D TOF-MRA (slice thickness 0.7 mm, TR 22 ms, TE 3.86 ms, 27.5% overlapping slices, flip angle 18°), and FLAIR (slice thickness 5 mm, TR 8000 ms, TE 100 ms; slice gap 0%). For CE-MRA (acquisition oriented in the coronal plane, slice thickness 0.95 mm, TR 4.16 ms, TE 1.44 ms, flip angle 20°), a fixed bolus of 5 ml Gadovist^®^ was administered at a flow rate of 5 ml/s, which was well tolerated in all patients reported.

Two different readers (first reader less than 1 year; second reader more than 3 year experience in stroke imaging) assessed SI on intracranial MRA. The measurements of the mean SI in the axial TOF-MRI were performed by placing regions of interest (ROI) within different parts of both ICAs using the scanner specific software tool. The surface areas ranged from 8 to 10 mm^2^. The cervical (C1) and petrous segment (C2) representing the extracranial part of the ICA and the lacerum (C3) and cavernous segment (C4) representing the intracranial part were chosen. Hereby, we provided four different ROIs on each side. We calculated a SI ratio (SIR) between the contralesional and lesional side according to the following formula: SIR = contralesional SI:lesional SI. The side of the stenosis found in US determined lesional SI. For the final analyses, we calculated one mean SIR from those of both readers.

### Statistical analysis

For statistical analysis, we used IBM SPSS Statistics 25. Categorical variables were compared using Chi-square test. We tested all continuous variables for normal distribution by Kolmogorov–Smirnov tests. Values for SI and patient’s age showed no normal distribution. To compare SIR between cases and controls in the primary case group and all subgroups, we used the Mann–Whitney *U* test. Receiver-operating characteristics (ROC) analysis was performed to determine a threshold value for the SIR with associated sensitivity and specificity. Interreader reliability was assessed using intraclass-correlation coefficients (ICC) to quantify the level of agreement regarding the SIR between readers. ICC estimates and their 95% confidence intervals were calculated based on mean-rating (*k* = 2), absolute-agreement and two-way mixed-effect model. In all analyses, a *p *value of < 0.05 (two-tailed) was used as a threshold for statistical significance.

## Results

### Patient characteristics

We screened 1201 digital patient records and excluded 1101 due to lack of extracranial ICA stenosis, extracranial occlusion, or bilateral carotid stenosis (see Fig. 1). In total, we included 51 patients (mean age 74; SD ± 9, 57% male) with middle-/high-grade extracranial ICAS without further vessel pathology into our primary case group. The secondary case group—subgroup A—consisted of 69 patients with middle-/high-grade extracranial ICAS including those with additional vessel pathology this time (mean age 73; SD ± 10, 59% male), as shown in Fig. 1.

The control group consisted of 100 patients without extracranial ICAS. Demographic and clinical information of the primary case group (*n* = 51) and controls (*n* = 100) are summarized in Table 1. Patients in the stenosis group (primary case group) were older and more likely to have ischemic stroke as the qualifying event than patients in the control group.

**Table 1:** Patients characteristics in the primary case group and control group

	Middle- or high-grade stenosis (*n* = 51) ^*^	No stenosis (*n* = 100)^*^	*p* value
Age, years (± SD)	74 (± 9)	68 (± 14)	**0.048**^†^
Male, *n* (%)	29 (57)	48 (48)	0.303^‡^
Acute stroke, *n* (%)	36 (71)	44 (44)	**0.002**^‡^
Hypertension, *n* (%)	46 (90)	82 (82)	0.288^‡^
Diabetes mellitus II, *n* (%)	13 (25)	21 (21)	0.822^‡^
Dyslipidemia, *n* (%)	45 (88)	88 (88)	0.999^‡^
Smoking, *n* (%)	14 (27)	24 (24)	0.311^‡^
Atrial fibrillation, *n* (%)	3 (6)	17 (17)	0.57^‡^

^*^Patients without additional vessel pathology (see main text for definition)

^†^Mann–Whitney *U *test was performed

^‡^Chi-square test was performed

Patients with middle-/high-grade extracranial ICAS were significantly older in comparison to the control group (74 vs. 68 years, *p* = 0.048) and had significantly more strokes (71% vs. 44%, *p* = 0.002). Cases and controls did not significantly differ with respect to sex and cardiovascular risk factors. To analyze if the differences in age between controls and cases exert any relevant effect on SIR, we dichotomized the control group (*n* = 100) in patients < 80 years (*n* = 78) and ≥ 80 years (*n* = 22). There were no significant differences in SIR between those groups (e.g., SIR-C3 1.043 vs. 1.064, *p* = 0.819; SIR-C4 1.033 vs.1.044, *p* = 0.876 for patients < 80 years and ≥ 80 years respectively).

The leading stroke cause in cases (*n* = 51) and controls (*n* = 100) was large-artery atherosclerosis as defined by the TOAST criteria [11] (88% vs 46%, *p* < 0.001); see supplementary data Table S3.

### Primary analysis

**Fig. 2:**
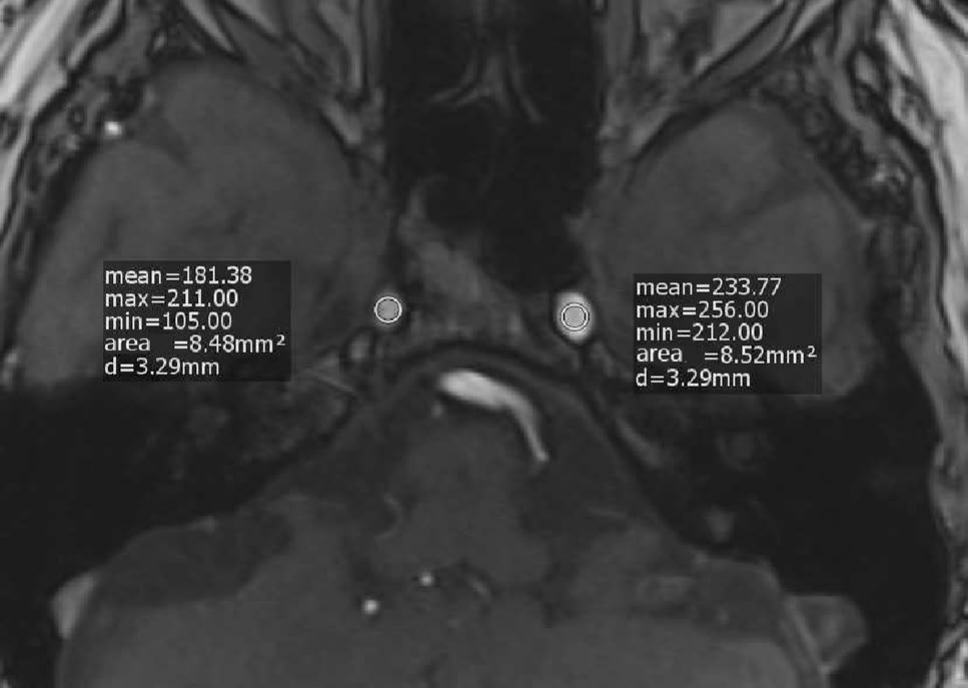
Measurement example for a patient with high-grade extracranial ICA stenosis on the right side. SI (signal inentsity) ratio = mean SI-contralesional:mean SI-lesional = 233.77:181.38 = 1.289

Our primary case group (*n* = 51) consisted of 24 patients with right-sided and 27 patients with left-sided extracranial ICAS. Compared to the control group, mean SIR differed significantly for right-sided ICAS in C3/C4 segments [1.463 (C4), *p* < 0.001; 1.613 (C3), *p* = 0.008] and for left-sided stenosis in C1–C4 segments [1.362 (C4), *p* < 0.001; 1.423 (C3), *p* < 0.001; 1.381 (C2), *p* = 0.002; 1.282 (C1), *p* = 0.011]. All results for the primary group are summarized in Table 2. A measurement example is given in Fig. 2.

**Table 2:** Signal intensity ratios (SIR) in cases and controls

	Stenosis (cases)^*^		No stenosis (control)^†^	
	Right^‡^	Left^§^	Right^‡^	Left^§^
No	24	27	100	
Mean SIR-C1 (± SD)	1.104 (± 0.66)	1.282 (± 0.45)	0.980 (± 0.15)	1.044 (± 0.15)
*p* value**	0.373	**0.011**		
Mean SIR-C2 (± SD)	1.163 (± 0.41)	1.381 (± 0.51)	0.993 (± 0.19)	1.040 (± 0.18)
*p* value**	0.057	**0.002**		
Mean SIR-C3 (± SD)	1.613 (± 0.95)	1.423 (± 0.54)	1.048 (± 0.24)	1.001 (± 0.24)
*p* value**	**0.008**	** < 0.001**		
Mean SIR-C4 (± SD)	1.463 (± 0.50)	1.362 (± 0.41)	1.035 (± 0.23)	1.000 (± 0.16)
*p* value**	** < 0.001**	** < 0.001**		

^*^Considering all patients with unilateral middle-/high-grade stenosis and without additional vessel pathology (see main text for definition)

^†^Considering all patients without any stenosis

^‡^SIR-right = SI-left/SI-right

^§^SIR-left = SI-right/SI-left

^**^Two-tailed Mann–Whitney *U* test comparing cases and controls was performed

The mean estimations of the intraclass-correlation coefficients along with 95% confidence intervals (CI) were excellent and showed good inter-rater reliability for SIR in all four segments on both sides [right side: ICC (C1) = 0.89 (CI_95%_ 0.85–0.92), ICC (C2) = 0.83 (CI_95%_ 0.78–0.87), ICC (C3) = 0.96 (CI_95%_ 0.95–0.97), ICC (C4) = 0.92 (CI_95%_ 0.89–0.94); left side: ICC (C1) = 0.96 (CI_95%_ 0.95–0.97), ICC (C2) = 0.94 (CI_95%_ 0.93–0.96), ICC (C3) = 0.96 (CI_95%_ 0.95–0.97, ICC (C4) = 0.97 (CI_95%_ 0.96–0.98)].

**Fig. 3:**
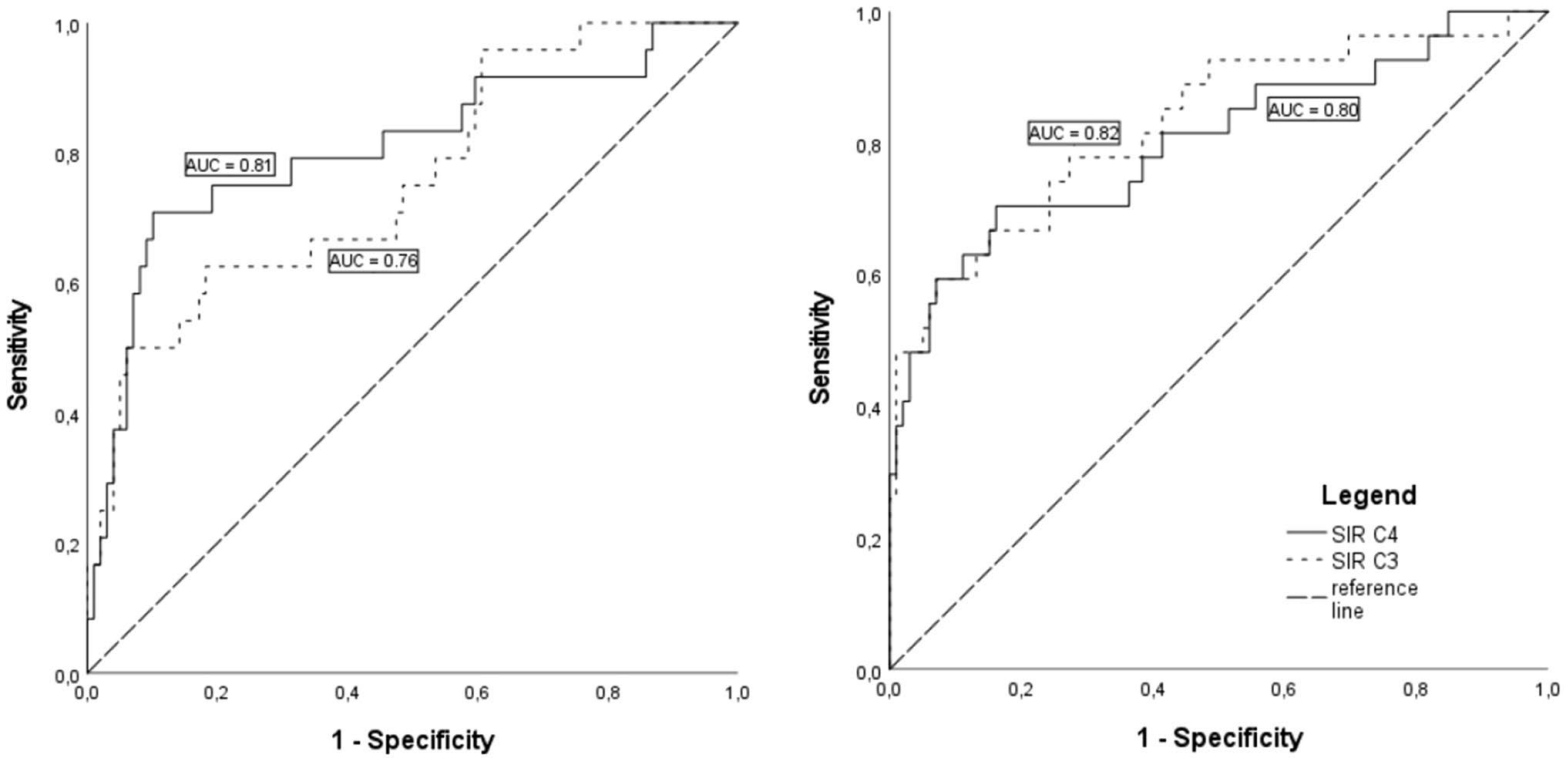
Receiver-operating characteristics (ROC) curve for signal intensity ratio (SIR) C3/C4. **A** Cases with right-sided middle-/high-grade stenosis without additional vessel pathology (*n* = 24); **B** cases with left-sided middle-/high-grade stenosis without additional vessel pathology (*n* = 27)

Summary ROC results are presented in Fig. 3.

ROC analyses showed an area under the curve (AUC) of 0.76 for C3 and 0.81 for C4 in case of right-sided stenosis (primary case group *n* = 24; control *n* = 100). Stenosis on the left side (primary case group *n* = 27; control *n* = 100) presented AUC of 0.82 for C3 and 0.80 for C4. A C4-SIR above 1.104 on the left side and 1.086 on the right side predicted a severe stenosis with a sensitivity/specificity of 70%/84% and 75%/81%, respectively.

### Secondary analysis

Regarding our secondary case group with additional vessel pathology—subgroup A—(*n* = 69), patients with right-sided stenosis (*n* = 34) showed a significant difference in SIR in C2–C4 compared to the control group (*n* = 100) [1.477 (C4), *p* < 0.001; 1.620 (C3), *p* = 0.002; 1.308 (C2), *p* = 0.034]. Patients with left-sided stenosis (n = 35) showed significantly increased SIR in all segments [(1.441 (C4), *p* < 0.001; 1.483 (C3), *p* < 0.001; 1.440 (C2), *p* < 0.001; 1.300 (C1), *p* = 0.001]; see supplementary data Table S1.

In patients with low-grade stenosis (< 50% according to NASCET), there was no significant difference in SIR in comparison to our control group, neither in subgroup B (with vessel pathology; *n* = 31), nor in subgroup C (without vessel pathology; *n* = 24); see supplementary data Table S1/S2.

## Discussion

In the present study, we demonstrated that unilateral middle- or high-grade extracranial ICAS results in significant increase of contralesional:lesional SIR in the intracranial parts of TOF-MRA. We could identify segments C3 and C4 of the ICA as best measuring points, as ROI positioning orthogonal to flow direction was easy to conduct due to their linear anatomy. In contrast, measurement in extracranial parts (C1 and C2) revealed mostly no significant differences. The same applied to low-grade stenosis. The findings were observed consistently in the subgroup analyses, suggesting that the results are applicable to patients with concomitant vessel pathologies, as well. ROC-derived cut-off levels of SIR obtained in C3 or C4 segments showed good discriminatory power to identify extracranial ICAS.

TOF-MRA enables the assessment of intracranial arteries using multiple repetitive radio frequency pulses to get stationary tissues magnetically saturated. Blood with unsaturated, fully magnetized protons entering the imaging volume generates a higher intraluminal signal than the adjacent stationary tissue with saturated protons [12, 13]. As SI correlates with the replacement of saturated spins, lower SI in TOF-MRA can indicate decreased flow, as in post-stenotic vessels, or increased random motion as in partial intraluminal obstruction [14].

This approach to use SI partly originates from US, which is often the first examination performed for diagnosing carotid stenosis. Flow velocity is one of the main parameters for evaluating the severity of carotid stenosis in Doppler US, and a ratio between the peak systolic velocity of the ICA and the common carotid artery was established, among other diagnostic parameters, for stenosis grading [15].

Against this background, we found that intracranial flow changes due to extracranial ICAS can be detected using TOF-MRA [5, 6]. Wu et al. already correlated SI ratios with the stenosis grade of extracranial ICAS [16]. While their standard of comparison was DSA, we used US as suggested by the European Stroke Organization (ESO) [17]. Moreover, they focused on stenoses grades of 80% or higher, whereas we included patients with ≥ 50% stenosis grade, as these patients—if symptomatic—benefit from carotid endarterectomy according to the guidelines of the ESO [18].

In neurovascular imaging, distal:proximal SI ratio in TOF-MRA is increasingly recognized as a non-invasive parameter to assess the hemodynamic impact of intracranial stenosis and has shown to indicate increased risk of stroke [19–24]. This approach is not promising when assessing extracranial ICAS in TOF-MRA. Alternatively, since flow in both ICAs is assumed to be equal in healthy persons, which is supported by our own data, we chose to use the unaffected side as comparison [25]. Another study already has shown that extracranial ICAS leads to significant reduction of the surface region distally by measuring the surface area and evaluating vessel asymmetry [26]. We consider C3/C4-SIR a more objective and investigator-independent method.

Unlike CE-MRA or CE-CTA, such flow-based parameter may provide additional information about a patient’s individual hemodynamics, especially in patients who undergo MRI in the first place. This could be the case in patients with less specific neurological symptoms (e.g., TIA or vertigo) and without known pre-existing cardiovascular conditions. In this setting, MRI offers the advantage of spotting even small ischemic lesions, and TOF-MRA can be used as a non-invasive screening method for extracranial ICAS. Once done, C3/C4-SIR can trigger further diagnostic measures, such as plaque imaging during the same MRI session. Especially, 3D black-blood MR imaging is becoming a preferred methodology for evaluating plaque burden and vulnerability non-invasively. Plaque morphology influences therapeutic decisions, since vulnerable plaques are associated with increased risk of ischemic stroke [27, 28]. After identifying patients with a possible extracranial ICAS US can verify and grade stenosis according to NASCET [10].

Limitations of our study are the following:

First, the present pilot study is a retrospective, non-blinded, single-center study with a relatively low sample size. Therefore, our findings need confirmation in a prospective and blinded validation study with larger sample sizes. While distal:proximal SIR in intracranial stenosis indicates worse outcome, such correlation with clinical outcomes is needed for the proposed parameter, as well. Furthermore, since neurovascular anatomy is highly variable and may influence hemodynamics and hereby SI in TOF-MRA, the influence of vessel malformations [29] and multiple vessel disease on contralesional:lesional SIR have to be studied in depth.

Second, case and control group were not balanced with respect to age and final diagnosis of stroke (versus TIA). This may have led to a bias, since the case group was significantly older than the control group. However, we argue that the difference in age is unlikely to distort the results of the study, since we found no significant differences in SIR between patients < 80 and ≥ 80 years after dichotomizing the control group.

Third, intrareader variability was not investigated in this study, but interreader reproducibility in SI measurement in TOF-MRA was high.

Fourth, disadvantages regarding the method may include the wide variance of SI depending on ROI positioning. Difficulties to recognize an appropriate measurement point arose in cases where the ICA caliber was highly reduced from stenosis. Bias was restricted by choosing relatively linear-flow segments, as C3/C4, where ROI positioning was easy to conduct. When discussing artifacts (e.g., turbulent flow, flow-related dephasing or susceptibility artifact from the sphenoid sinus), typical for the distal ICA in TOF-MRA, one should consider that they occur most likely in both ICAs and by including both sides in the SI ratio bias was minimized.

Finally, ultrasound was performed by different examiners, leading to potential differences in stenosis evaluation.

## Conclusion

Contralesional:lesional SIR in C3/C4 on axial intracranial TOF-MRA can be used as an additional contrast-agent free method to recognize relevant unilateral extracranial ICAS in patients with acute ischemic stroke, TIA, or unspecific neurological symptoms.

## Supplementary Information

Below is the link to the electronic supplementary material.

Supplementary file1 (DOCX 26 KB)

## Data Availability

Not applicable.
