# Supplementary material for: Can intracranial time-of-flight-MR angiography predict extracranial carotid artery stenosis?
Source: J Neurol. 2021 Nov 9;269(5):2743–9. doi: 10.1007/s00415-021-10876-0 (PMC9021057; doi:10.1007/s00415-021-10876-0)
Supplement: Supplementary file 1 — Supplementary file1 (DOCX 26 KB) [file 415_2021_10876_MOESM1_ESM.docx]

**Journal of Neurology**

**Supplementary Material**

**Can intracranial Time-of-flight-MR Angiography predict extracranial carotid artery stenosis?**

**Filiz Osmanodja^1,3^, Jan F Scheitz^1,2^, MD, Jochen B Fiebach^1^, Ramanan Ganeshan^1,2^, MD, Kersten Villringer^1^, MD**

1 Center for Stroke Research Berlin (CSB), Charité – Universitätsmedizin Berlin, corporate member of Freie Universität Berlin, Humboldt-Universität zu Berlin, and Berlin Institute of Health, Berlin, Germany.

2 Department of Neurology, Charité – Universitätsmedizin Berlin, corporate member of Freie Universität Berlin, Humboldt-Universität zu Berlin, and Berlin Institute of Health, Berlin, Germany.

3 Department of Neuroradiology, Friedrich-Alexander-University Erlangen-Nürnberg, Schwabachanlage 6, 91054 Erlangen, Germany.

**Correspondence:**  Filiz Osmanodja
Department of Neuroradiology, Friedrich-Alexander-University Erlangen- Nürnberg, Schwabachanlage 6, 91054 Erlangen, Germany. Phone +49-913185044844, filiz.osmanodja@uk-erlangen.de

Table S1: Signal Intensity ratios (SIR) in cases and controls (Subgroup A/B: with or without vessel pathology)

|  | Stenosis (case) ^*^ | | | | No stenosis (control) ^†^ | |
| --- | --- | --- | --- | --- | --- | --- |
|  | Right^‡^ | | Left^§^ | | Right^‡^ | Left^§^ |
|  | lowgrade stenosis | middle/ highgrade stenosis | lowgrade stenosis | middle/ highgrade stenosis |  |  |
| N. | 15 | 34 | 16 | 35 | 100 | 100 |
| mean SIR-C1 (± SD) | 1.003 (± 0.21) | 1.114 (± 0.58) | 1.108 (± 0.18) | 1.300 (± 0.41) | 0.980 (± 0.15) | 1.044 (± 0.15) |
| p-value^**^ | 0.445 | 0.197 | 0.396 | **0.001** |  |  |
| Mean SIR-C2 (± SD) | 1.006 (± 0.20) | 1.308 (± 0.82) | 1.105 (± 0.22) | 1,440 (± 0,52) | 0.993 (± 0.19) | 1.040 (± 0.18) |
| p-value^**^ | 0.836 | **0.034** | 0.126 | **< 0.001** |  |  |
| mean SIR-C3 (± SD) | 0.957 (± 0.16) | 1.620 (± 1.00) | 1.055 (± 0.19) | 1.483 (± 0.61) | 1.048 (± 0.24) | 1.001 (± 0.24) |
| p-value^**^ | 0,259 | **0.002** | 0,302 | **< 0.001** |  |  |
| mean SIR-C4 (± SD) | 0.976 (± 0.16) | 1.477 (± 0.60) | 1.089 (± 0.17) | 1.441 (± 0.53) | 1.035 (± 0.23) | 1.000 (± 0.16) |
| p-value^**^ | 0.943 | **< 0.001** | 0.137 | **< 0.001** |  |  |
| ^*^ Considering all patients with unilateral stenosis  ^†^ Considering all patients without any stenosis  ^‡^ SIR-right = SI-left/ SI-right  ^§^ SIR-left = SI-right / SI-left  ^**^ t- test comparing cases and controls was performed | | | | | | |

Table S2: Signal Intensity ratios (SIR) in cases and controls (Subgroup C: low-grade stenosis without vessel pathology)

|  | Stenosis (case) ^*^ | | No stenosis (control) † | |
| --- | --- | --- | --- | --- |
|  | Right^‡^ | Left^§^ | Right^‡^ | Left^§^ |
| N. | 11 | 13 | 100 | |
| mean SIR-C1 (± SD) | 1.040 (± 0.21) | 1.051 (± 0.10) | 0.980 (± 0.15) | 1.044 (± 0.15) |
| p-value^**^ | 0.164 | 0.971 |  |  |
| mean SIR-C2 (± SD) | 1.034 (± 0.22) | 1.080 (± 0.21) | 0.993 (± 0.19) | 1.040 (± 0.18) |
| p-value^**^ | 0.657 | 0.268 |  |  |
| mean SIR-C3 (± SD) | 0.982 (± 0.17) | 1.040 (± 0.20) | 1.048 (± 0.24) | 1.001 (± 0.24) |
| p-value^**^ | 0.693 | 0.666 |  |  |
| mean SIR-C4 (± SD) | 0.998 (± 0.15) | 1.060 (± 0.17) | 1.035 (± 0.23) | 1.000 (± 0.16) |
| p-value^**^ | 0.587 | 0.564 |  |  |
| ^*^ Considering all patients with unilateral low-grade stenosis and without vessel pathology (see text for definition)  ^†^ Considering all patients without any stenosis  ^‡^ SIR-right = SI-left/SI-right  ^§^ SIR-left = SI-right/SI-left  ^**^ Two-tailed Mann-Whitney-U test comparing cases and controls was performed | | | | |

|  | Stenosis (n=51) ^*^ | No stenosis (n=100) ^†^ | p-value |
| --- | --- | --- | --- |
| Large-artery atherosclerosis^§^, n (%) | 45 (88) | 46 (46) | **<0.001**^‡^ |
| Cardioembolism^§^, n (%) | 0 (0) | 18 (18) | **<0.001**^‡^ |
| Small-vessel occlusion^§^, n (%) | 3 (6) | 8 (8) | 0.636^‡^ |
| Stroke of other determined etiology^§^, n (%) | 0 (0) | 1 (1) | 0.474^‡^ |
| Stroke of undetermined etiology^§^, n (%) | 1 (2) | 11 (11) | 0.052^‡^ |
| Other diagnosis^§^, n (%) | 2 (4) | 16 (16) | **0,030**^‡^ |
| Hypoplastic A1-segment, n (%) | 6 (12) | 14 (14) | 0,925^‡^ |
| Fetal posterior cerebral artery, n (%) | 15 (29) | 28 (28) | 0,429^‡^ |
| ^*^ Considering all patients with unilateral middle-/high-grade stenosis and without additional vessel pathology (see main text for definition)  ^†^ Considering all patients without any stenosis  ^‡^ χ2 test was performed  ^§^ according to the Trial of ORG 10172 in Acute Stroke Treatment (TOAST) criteria [1] | | | |

Table S3: Stroke etiology and variant anatomy of the circle of willis in cases and controls

1. Adams, H.P., Jr., et al., *Classification of subtype of acute ischemic stroke. Definitions for use in a multicenter clinical trial. TOAST. Trial of Org 10172 in Acute Stroke Treatment.* Stroke, 1993. **24**(1): p. 35-41 DOI: 10.1161/01.str.24.1.35.
